# Hypertension trials update

**DOI:** 10.1038/s41371-020-00477-1

**Published:** 2021-01-12

**Authors:** Hussam Al Ghorani, Saarraaken Kulenthiran, Lucas Lauder, Michael Böhm, Felix Mahfoud

**Affiliations:** Klinik für Innere Medizin III, Kardiologie, Angiologie und Internistische Intensivmedizin, Universitätsklinikum des Saarlandes, Saarland University, Homburg, Germany

**Keywords:** Renovascular hypertension, Risk factors

## Abstract

Hypertension is one of the most prevalent cardiovascular diseases and its treatment requires multimodal therapeutic approaches. This review aims to provide a summary and update on relevant evidence in hypertension research published in 2019/2020. These include trials dealing with the prognostic effect of systolic and diastolic blood pressure values, the association between hypertension and valve disease, reproducibility of masked and white-coat hypertension, and the prognostic importance of ambulatory and night-time blood pressure measurements. Treatment of hypertension focusing on elderly patients but also the potential cancer risk of thiazide diuretics, the valsartan recall, chronotherapy, and device-based hypertension therapy are discussed.

## Introduction

Arterial hypertension is the most frequent modifiable risk factor associated with cardiovascular morbidity and mortality [1]. Despite the widespread availability of proven approaches to lower blood pressure (BP), i.e. lifestyle modification and pharmacotherapy, in many patients BP control to guideline recommended targets is not achieved [2]. This review aims at critically discussing and summarizing relevant trials in hypertension research, which were published recently in major journals. We discuss, among others, the independent association of elevated systolic (SBP) and diastolic BP (DBP) with cardiovascular (CV) outcomes, and the prognostic relevance of 24-h and night-time BP measurements for estimation of CV Risk. Current guidelines have recommended lowering SBP in most patients (<65 years) to targets of 120–130 mmHg. The treatment targets in patients of older age and the neutral effects of lowering BP ≤ 140/90 mmHg in these patients are reviewed. The Hygia trial investigated the impact of bedtime versus morning dosing and found significant improvements of CV morbidity and mortality associated with bedtime administration of antihypertensive medication. However, there remain major limitations to be considered before this trial should change clinical practice. Finally, the most recent trials in device-based hypertension treatments, above all the results of the first pivotal study of catheter-based renal denervation, are presented.

### Epidemiology und pathophysiology

#### The Effect of systolic and diastolic blood pressure on cardiovascular outcomes

**Fig. 1:**
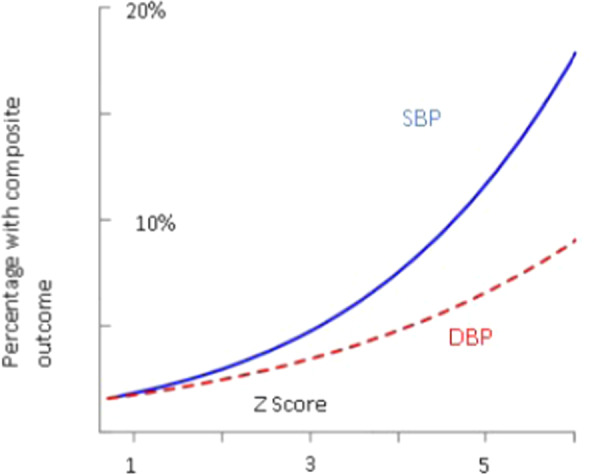
The effect of systolic and diastolic blood pressure on cardiovascular outcomes. The relationship between systolic blood pressure (solid line) and diastolic blood pressure (dashed line) *z* scores and the risk of the composite outcome among participants with systolic blood pressure > 133 mmHg or diastolic blood pressure > 78 mmHg are shown [3].

High SBP and DBP associate with increased CV events. However, it remained unclear whether either SBP or DBP carries more prognostic information. This question was addressed in a study, which evaluated data from an integrated health care delivery system in the US. Between 2007 and 2016, more than 36 million office BP measurements were analyzed from around 1.3 million people [3]. The study confirmed that both SBP and DBP increased the risk of CV events such as myocardial infarction and stroke. A continuous burden of SBP (≥140 mmHg; hazard ratio (HR) per unit increase in z score, 1.18; 95% confidence interval [CI], 1.17 to 1.18) and DBP (≥90 mmHg; HR per unit increase in z score, 1.06; 95% CI, 1.06 to 1.07) independently predicted the composite outcome. A J-curve relationship between DBP and the composite outcome was detected. Elevated SBP had a greater prognostic effect on stroke and myocardial infarction (Fig. 1). However, DBP influenced the prognosis independently of SBP.

**Fig. 2:**
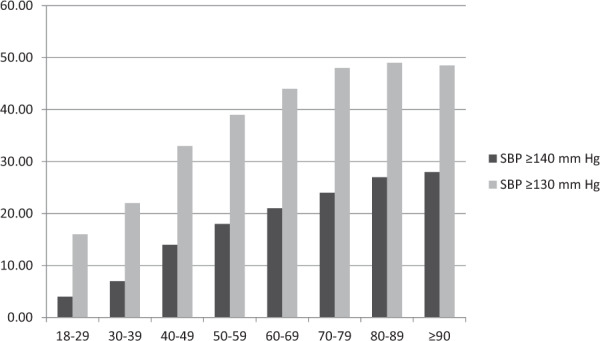
Prevalence of systolic blood pressure ≥130 or ≥140 mmHg depending on age. Systolic blood pressure measurements indicating hypertension (systolic blood pressure ≥ 130 mmHg or ≥140 mmHg) increased as a function of age [3].

The 2017 American College of Cardiology and American Heart Association Guidelines on the management of hypertension revised the definition of hypertension [4], which has been defined as >130/>80 mmHg. Contrary, in the 2018 European Guidelines, the definition remained unchanged (>140/>90 mmHg) [2]. Against this background, the prevalence of hypertension according to the different definition criteria were analyzed. The prevalence of hypertension increased from 18.9% with a threshold of >140/90 mmHg to 43.5% with >130/80 mmHg. An SBP of >130 mmHg was present in almost 50% of the participants over the age of 60 years (Fig. 2).

##### Clinical implication

SBP and DBP are independently associated with myocardial infarction and stroke, although SBP has a greater prognostic effect. The J-curve relationship between DBP and the composite outcome was strongly influenced by age and coexisting conditions such as high SBP, diabetes, coronary disease, smoking, and history of stroke.

#### Hypertension and heart valve disease

**Fig. 3:**
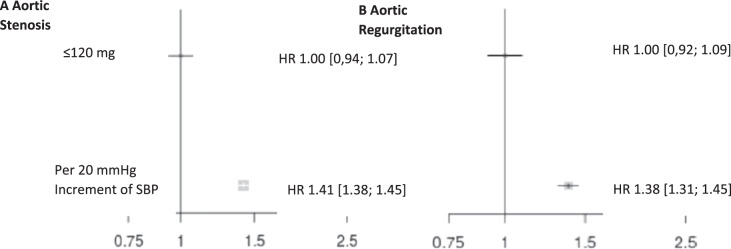
The effect of systolic blood pressure on aortic valve diseases. Shown are hazard ratios for aortic stenosis (**a**) and aortic regurgitation (**b**) by categories of systolic blood pressure. Hazard ratios (HR) and 95% confidence intervals (CI) are displayed using floating absolute risk. Square sizes are inversely proportional to standard error and horizontal lines depict 95% confidence intervals. Models are adjusted for age, sex, body mass index, smoking, year of initial blood pressure measurement, total cholesterol, LDL, HDL, and practice-level index of multiple deprivation [6].

**Fig. 4:**
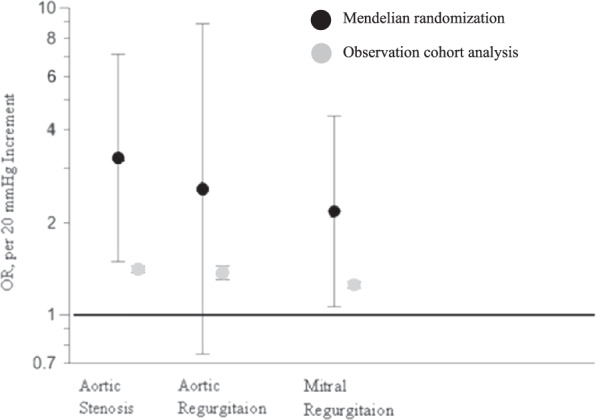
Association between systolic blood pressure increment and valvular heart disease outcomes. The figure shows an increase in the odds ratio per 20 mmHg increment in systolic blood pressure. Circles show point estimation and vertical lines represent 95% CIs. Odds ratio indicates hazard ratio in observational cohort studies and odds ratio in mendelian randomization estimation. Data collected from observation analyses and genetic studies [7].

Hypertension not only increases the risk of stroke, myocardial infarction, and renal failure [5] but also appears to be associated with heart valve diseases such as aortic valve stenosis and aortic valve insufficiency [6]. In a study from the UK, 5.4 million patients with unknown CV disease or aortic valve disease at baseline were examined and followed for 9.2 years [6]. A total of 20,680 patients (0.38%) were diagnosed with aortic valve stenosis and 6440 patients (0.12%) with aortic regurgitation. For each increase of 20 mmHg in SBP, the relative risk [RR] of aortic valve stenosis increased by 41% (HR 1.41, 95% CI 1.38–1.45), and 38% for aortic regurgitation (HR: 1.38, CI 1.31–1.45) (Fig. 3), respectively. These findings are associations and do not prove causation. Therefore, a study which utilized Mendelian randomization methods analyzed data from 329,237 gene-typed subjects from the UK (40–96 years of age). Mendelian randomization is a method based on the natural distribution of gene variations associated with certain diseases to investigate the association between a risk factor and a disease [7]. This method can be used to randomize participants based on polymorphisms associated with lifelong exposure to influenceable risk factors—in this case increased SBP. If an association between the polymorphisms and the disease can be demonstrated, this strongly suggests a causal relationship between exposure and disease. The participants were recruited between 2006 and 2010. A total of 130 gene variants were selected for the genetic determination of hypertension. A total of 1491 (0.45%) patients had aortic valve stenosis, 634 (0.19%) aortic regurgitation and 1736 (0.53%) mitral regurgitation. With each increase in SBP by 20 mmHg, the RR for aortic valve stenosis increased by 3.3-fold (odds ratio [OR] 3.26; 95% CI 1.50–7.10, *p* = 0.002), for aortic regurgitation by 2.5 (OR 2.59; 95% CI 0.75–8.92, *p* = 0.13), and for mitral valve regurgitation by 2.2 (OR 2.19; 95% CI 1.07–4.47, *p* = 0.03), respectively. An increase in SBP by 20 mmHg was associated with an almost 3-fold higher risk to develop any of the three valvular diseases (OR 2.85; 95% CI 1.69–4.78, *p* < 0.001) (Fig. 4). These data suggest that lifetime exposure to elevated SBP appears to be associated with an increased risk of major valvular heart disease.

##### Clinical implication

The studies show an association between hypertension and valvular heart disease outcomes. It remains to be seen how BP control may affect the incidence of heart valve disease progression.

### Blood pressure measurement and target blood pressure

#### Prognostic value of ambulatory blood pressure monitoring and night-time blood pressure

**Fig. 5:**
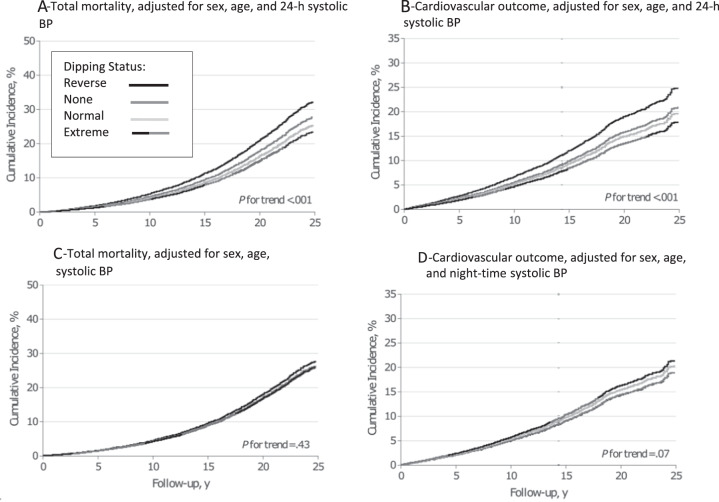
Incidence of mortality and cardiovascular outcomes, adjusted for gender, age, nighttime and 24-h BP. Shown is the incidence of mortality, adjusted for sex, age and 24-h systolic blood pressure (**A**), cardiovascular outcomes, adjusted for sex, age and 24-h systolic blood pressure (**B**), total mortality, adjusted for sex, age and night-time systolic blood pressure (**C**) and cardiovascular outcomes, adjusted for sex, age and night-time systolic blood pressure (**D**) over follow-up in years based on systolic dipping ratio [8].

The 2018 European Guidelines on the management of arterial hypertension [2] recommend that the diagnosis of hypertension should not only be dependent on office BP measurements but also on “out-of-office” measurements, such as ambulatory blood pressure monitoring (ABPM) or home blood pressure monitoring (HBPM). A study with 11,135 adults aged 54.7 years (49% women) from 13 cohorts investigated the association between mortality and CV outcomes with the different methods of BP measurement (office and conventional BP measurements, automated measurements, 24-h SBP, day- and night-time BP) [8]. The primary endpoints were mortality and a composite of CV events consisting of cardiovascular mortality, non-fatal cardiovascular events, heart failure, and stroke. The average follow-up was 13.8 years, 43.7% of patients had high BP (defined as >140/90 mmHg), 46.5% were on antihypertensive drugs. A total of 2,836 (18.5 per 1000 person-years) participants died and 2,049 (13.8 per 1000 person-years) experienced a CV event. Both endpoints were significantly associated with all single SBP indexes [8] (*p* < 0.001). Higher 24-h and night-time BP measurements were significantly associated with greater risks of death and the composite CV outcome (Table 1, Fig. 5). For night-time SBP, the HR for mortality was 1.23 (95% CI, 1.17–1.28) and 1.36 (95% CI, 1.30–1.43) for CV events. For 24-h SBP, the HR for mortality was 1.22 (95% CI. 1.16–1.28) and 1.45 (95% CI, 1.37–1.54) for CV events. In models that adjusted for any of the other SBP indexes, the association of night-time and 24-h SBP with the primary outcome remained statistically significant (HRs ranging from 1.17 (95% CI, 1.10–1.25) to 1.87 (95% CI, 1.62–2.16)). For every 20/10 mmHg increment of BP measured at night, the risk of mortality increased by 23% and the risk of cardiovascular events by 36%.

**Table 1:** Association of outcomes with 24 h or night-time SBP Adjusted for other SBP Indexes [8].

	SBP			
	24 h		Night-time	
Outcomes	Hazard Ratio (95% CI)	*P* Value	Hazard Ratio (95% CI)	*P* value
**Total Mortality (*n*** = **2836)**				
SBP index				
Conventional	1.17 (1.10–1.25)	<0.001	1.20 (1.14–1.26)	<0.001
Automated office SBP	1.25 (1.17–1.34)	<0.001	1.22 (1.17–1.29)	<0.001
Measure times				
24 h	NA	NA	1.24 (1.14–1.36)	<0.001
Daytime	1.87 (1.62–2.16)	<0.001	1.24 (1.17–1.31)	<0.001
Nighttime	1.25 (1.11–1.41)	<0.001	NA	NA
Dipping ratio	1.43 (1.34–1.51)	<0.001	1.21 (1.14–1.28)	<0.001

##### Clinical implication

24-h and night-time BP measurements were associated with greater risks of mortality and a composite of CV outcome. Thus, they may be considered as the most relevant measurements for estimating CV risk.

#### Reproducibility of masked and white-coat hypertension

**Fig. 6:**
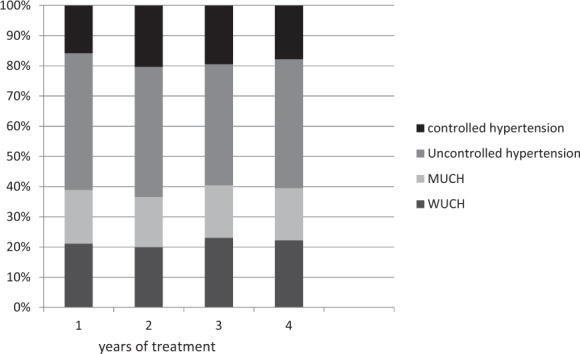
Prevalence of different hypertensive phenotypes over the 4-year follow-up period. Masked uncontrolled hypertension (MUCH), white-coat uncontrolled hypertension (WUCH), controlled hypertension, and uncontrolled hypertension are shown as % values during the 4 years of antihypertensive treatment in the European Lacidipine Study on Atherosclerosis trial [10].

**Fig. 7:**
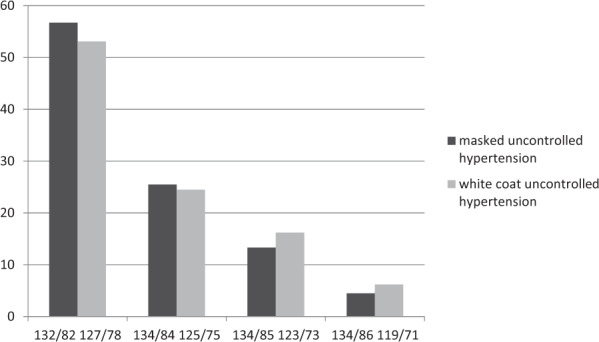
Proportion of patients diagnosed with masked or white coat hypertension during the 4-year follow-up period. The percentage of these patients decreases over time, which indicates a limited reproducibility by frequently blood pressure measurements [10].

Ambulatory BP measurements are essential for the detection of masked (normal office BP values, increased ambulatory BP values) and white-coat hypertension (increased office BP values, normal ambulatory BP values). The reproducibility of these BP phenotypes, however, is not well known. In an analysis of 1664 hypertensive patients who were treated with atenolol or lacidipine for four years as part of the European Lacidipine Study on Atherosclerosis (ELSA) [9], the reproducibility of masked and white-coat hypertension was examined [10]. Both office and 24-h BP  were measured at baseline and every year of the 4-year follow-up. After one year of treatment, the prevalence of masked hypertension and white-coat hypertension was 21.1% and 17.8%, respectively (Fig. 6). The prevalence of the phenotypes remained relatively constant throughout the follow-up period, but the population of the cohort changed significantly. Only about one-third of patients classified as masked or white-coat hypertension according to one set of office and ambulatory BP measurements maintained the same classification at a subsequent set of measurements. The number of patients diagnosed with masked or white-coat hypertension over the entire observation period was 4.5% and 6.2% only (Fig. 7).

##### Clinical implication

The overall reproducibility of masked and white-coat hypertension is low and suggests that regular BP examinations (using different measurement modalities) are necessary.

#### Target blood pressure values in elderly patients

According to recent guidelines, treatment of hypertension in patients aged over 80 years should be initiated when BP is ≥160/≥90 mmHg (see Table 2) [2]. The 2018 European Guidelines [2] recommend a BP treatment target of <130/<80 mmHg in patients under 65 years of age [2]. The DBP target is generally <80 mmHg regardless of age or comorbidities. In contrast to previous guidelines, the current guidelines recommend that SBP should be targeted to a BP range of 130–139 mmHg. These recommendations are based on evidence indicating that the lowest risk of CV events (especially cardiovascular death and congestive heart failure) is observed at these values [11–13]. The 2018 European Guidelines emphasize that these values may even be applied to patients over 80 years, provided that an SBP between 130 and 140 mmHg is well tolerated.

**Table 2:** Office blood pressure thresholds for treatment [15].

Age group	Office SBP treatment threshold (mmHg)					Office DBP treatment threshold (mmHg)
	Hypertension	+ Diabetes	+ CKD	+ CCS	+ Stroke/TIA	
18–65	≥140	≥140	≥140	≥140	≥140	≥90
65–79	≥140	≥140	≥140	≥140	≥140	≥90
≥80	≥160	≥160	≥160	≥160	≥160	≥90
Office DBP treatment threshold (mmHg)	≥90	≥90	≥90	≥90	≥90	

**Fig. 8:**
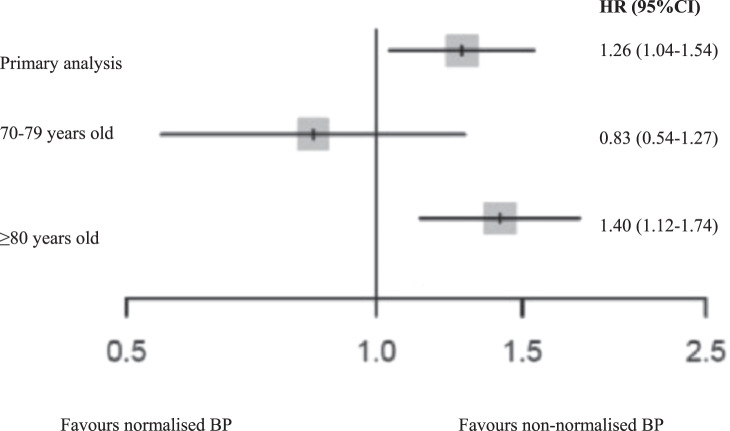
Primary and stratified analyses of participants. Forest-plot summarizing the primary analysis and the stratified analyses for blood pressure values <140/90 or >140/90 mmHg [14].

The Berlin Initiative Study [14] examined the influence of BP lowering in patients older than 70 years. The study included 1628 patients with a mean age of 81 years who were treated with antihypertensive drugs (636 exhibited normalized BP < 140/<90 mmHg). Patients with a BP < 140/90 mmHg had a 26% higher risk of all-cause mortality (HR 1.26; 95% CI 1.04–1.54) than those whose BP above. The relative risk was further increased by 40% in patients ≥80 years (HR 1.40; 95% CI 1.12–1.74), and likewise in patients with previous CV events (HR 1.61; 95% CI 1.14–2.27). However, this risk amplification was not observed in patients between 70 and 79 years (HR 0.83; 95% CI 0.54–1.27). The increased risk in the elderly was particularly influenced by SBP values <130 mmHg (Fig. 8). The association between SBP and mortality demonstrated in a U-shaped curve using 140 mmHg as reference. There was an increased risk with lower SBP values that reached statistical significance at 125 mmHg, while the numerically increased risk with higher SBP values did not reach statistical significance.

##### Clinical implication

BP values ≤140/90 mmHg may be associated with an increased risk of mortality in octogenarians or elderly patients with previous cardiovascular events. The risk-benefit assessment of an intensified BP treatment should be critically and individually evaluated, especially in older patients.

#### Medical Therapy

Most patients with hypertension require lifelong medical therapy to achieve optimal BP control. The 2018 European Guidelines [2] equally recommend five classes of antihypertensive drugs; these include ACE inhibitors (ACE-I), angiotensin receptor blockers (ARB), beta blockers (BB), calcium channel blockers (CCB), and diuretics (thiazides and thiazide-like diuretics) [2].

**Fig. 9:**
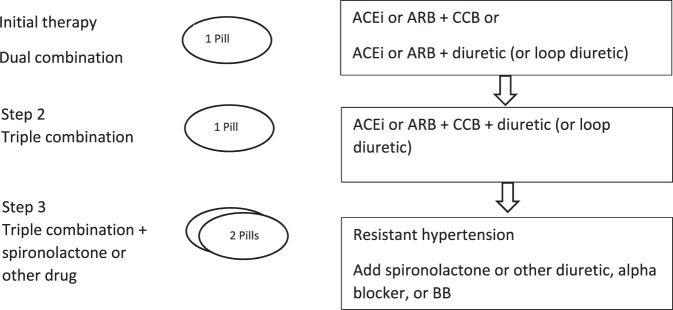
Treatment strategy for uncomplicated hypertension. ACEi angiotensin-converting enzyme inhibitor, ARB angiotensin receptor blocker, CCB calcium channel blocker [2].

Given the high rates of non-adherence to antihypertensive medication, the treatment regime should be effective and above all uncomplicated [15]. Hence, the importance of combination treatment is particularly highlighted to improve adherence to therapy and BP control. Therefore, the 2018 European Guidelines [2] recommend, especially in the context of lower BP targets, to start antihypertensive therapy with an initial dual fixed-dose combination of ACE-I or ARB + CCB or diuretic (see Fig. 9). These combinations should be the primary therapy for most patients. Exceptions are patients with grade 1 hypertension (especially with SBP values <150 mmHg), low risk as well as elderly (≥80 years of age) or frail patients. If BP is not controlled with a two-drug combination, it is recommended escalating to a three-drug combination of ACE-I or ARB + CCB + diuretic (i.e. a triple combination) (see Fig. 9). If BP still remains uncontrolled, despite triple therapy in sufficient doses, treatment should be increased by addition of spironolactone 25–50 mg or, if not tolerated, other diuretics such as amiloride or higher doses of other diuretics, a BB, or an alpha-blocker should be added (see Fig. 9). Therapy with BB is recommended especially for patients with cardiac comorbidities (heart failure, angina pectoris, post-myocardial infarction, atrial fibrillation, or younger women).

#### Thiazide—the first-choice antihypertensive drug?

The LEGEND project (Large-Scale Evidence Generation and Evaluation across a Network of Databases) evaluated data of 4.9 million patients from 9 different databases in the USA, South Korea, Germany and Japan, in which treatment of hypertension was initiated with a single drug [16]. A total of 55 safety and efficacy endpoints were examined. The following three events were chosen as primary endpoints: acute myocardial infarction, heart failure hospitalization and stroke. Thiazide and thiazide-like diuretics showed better primary effectiveness compared with ACE-I. The relative risk reduction when treated with thiazide diuretics compared with ACE-I was 17% (HR 0.83; 95% CI 0.74–0.95) for stroke, 17% (HR 0.83; 95% CI 0.74–0.95) for heart failure hospitalization and 16% (HR 0.84; 95% CI 0.75–0.95) for acute myocardial infarction, respectively. There were no differences between ACE-I, ARB and CCB. Significantly inferior to all classes were non-dihydropyridine-CCB (diltiazem). Interestingly, the side effects were comparable across all groups.

##### Clinical implication

One of the limitations of this analysis is the retrospective design. Physicians might have preferred ACE-I over a diuretic in patients with increased CV risk. That would explain why the effects on CV diseases in this group are more significant compared with the ACE-I-group. Furthermore, patients were treated with monotherapy, which is no longer recommended. Lastly, information about adherence to therapy and BP data are missing.

#### Hydrochlorothiazide and the increased the risk of cancer

Hydrochlorothiazide (HCT) is rarely used as a monotherapy but is a frequent component of double or triple combination preparations. The effectiveness of HCT compared with other thiazide-like diuretics has been the subject of controversial discussions [2, 17]. The warning published by the European Medicines Agency (EMA) and the Federal Institute for Drugs and Medical Devices in Germany regarding the potential risk of basal cell carcinoma (BCC) and squamous cell carcinoma (SCC) of skin should be considered when prescribing HCT-containing drugs [18]. This letter was triggered by data from the Danish Cancer Registry and the National Prescription Registry. These studies from Denmark showed a cumulative dose-related relationship between HCT and non-melanoma skin cancer (NMSC) [19, 20]. The Danish study documented a dose-dependent association between HCT and the risk for BCC, ranging between 1.29 (95%CI 1.23–1.35) times for ≥50 g of HCT and 7.7 (95%CI 5.7–10.5) times for ≥100 g of HCT for SCC. As with any cohort study, only an association can be proven, but no causality. There is also a lack of information on the sun exposure of the patients and data on the aggressiveness of the neoplasia.

##### Clinical implication

The Drug Commission of the German Medical Association does not recommend a general change in therapy for all HCT-treated patients, but individual examination and regular skin inspections. Chlorthalidone and indapamide are possible alternatives, but no data are available on the potential risk for skin cancer of these thiazide-like diuretics [21].

#### Valsartan recall

Since early July 2018, products containing valsartan have been recalled worldwide. The reason is the detection of a known carcinogen, namely N-nitrosodimethylamine (NDMA), which can be found in candesartan, irbesartan, losartan, olmesartan, and valsartan. NDMA has been classified by the WHO International Agency for Research on Cancer to be carcinogenic in humans [22]. The recall of drugs containing valsartan from two Chinese manufacturers that were contaminated with NDMA affected around 40% of patients treated with valsartan in Germany. In the risk assessment process, the EMA used a conservative assessment of the possible cancer risk and came to the following conclusion: If 100,000 patients would have received NDMA-contaminated valsartan from Zhejiang Huahai (manufacturing site where the highest levels of contaminants were found) every day for 6 years in the highest dose, it could result in 22 additional liver cancers over the lifetime of these patients. The presence of NDMA in these drugs could lead to 8 additional cancer cases in 100,000 patients if they had taken the highest daily dose over 4 years [23].

**Fig. 10:**
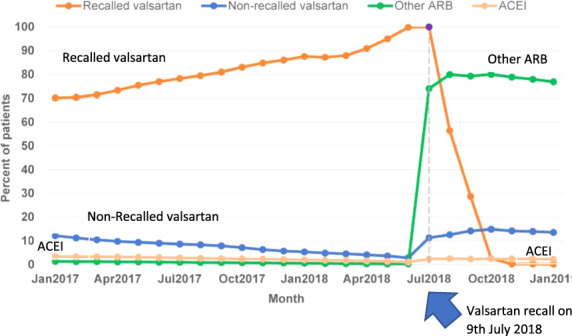
Prescribing behavior after the Valsartan recall in Canada. Most patients (74%) were switched to a different ARB (not 357 valsartan). No alternative antihypertensive drug was prescribed in 11% of the patients [24].

A study from Canada investigated the consequences of the valsartan recall in more detail [24]. The study included 55,461 patients with a mean age of 76 years, 41% were male, 95% had hypertension while 5% had heart failure. Most patients (74%) were switched to a different ARB (not valsartan). No alternative antihypertensive drug was prescribed in 11% of the patients. This was accompanied by a significant increase in the rate of emergency department visits of +6% (*p* = 0.020) and hospitalization for stroke/TIA of 8% (*p* = 0.037) (see Fig. 10) [24].

##### Clinical implication

Due to the recall/warning regarding valsartan, a relevant number of patients did not fill an alternative prescription. Consequently, the number of patients with unsatisfactory BP control and hospitalization for hypertension and stroke/TIA increased.

#### Patiromer and spironolactone in the therapy of resistant hypertension

Approximately 5–15% of all hypertensive patients have resistant hypertension, which is associated with a significantly increased CV risk [25]. Resistant hypertension is defined as the failure of conservative treatment strategies to lower office SBP and DBP values to <140 mmHg and/or <90 mmHg despite optimal or best-tolerated doses of three or more drugs, which should include a diuretic.

The PATHWAY-2 [26] trial was double-blind, placebo-controlled, crossover trail, that compared different BP-lowering medications in 335 patients with resistant hypertension. In a preassigned, randomized order, participants were treated for 12 weeks each with spironolactone (25–50 mg), bisoprolol (5–10 mg), doxazosin modified release (4–8 mg), and placebo, in addition to their baseline BP medication. During the first six weeks of each treatment cycle, the drugs were initiated at the lower dose. After that, the drug doses were doubled for the second half of the treatment cycle. The average reduction in home SBP was greater with spironolactone than with placebo (−8.7 mmHg; 95% CI −9.7 to −7.7; *p* < 0.0001), doxazosin (−4.0 mmHg; 95% CI −5.0 to −3.0; *p* < 0.0001) or bisoprolol (−4.5 mmHg; 95% CI −5.5 to −3.5; *p* < 0.0001). The side effect profile of the substances was comparable over the 3-month investigation period. Thus, spironolactone has been recommended as a fourth-line therapy in patients with uncontrolled hypertension. The use of spironolactone, however, is often limited by hyperkalemia, which is associated with increased mortality.

**Fig. 11:**
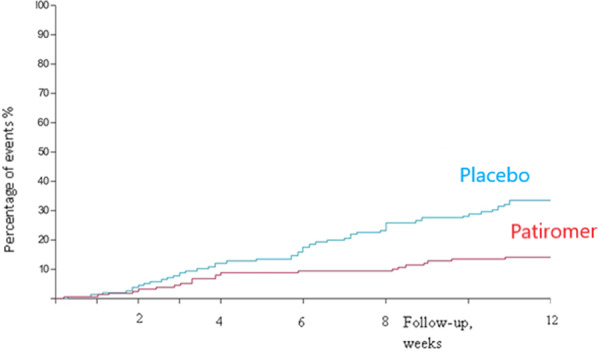
Time to discontinuation of spironolactone in patients with and without patiromer. The proportion of patients who did not have to discontinue spironolactone was higher in patients receiving patiromer compared with those receibing a placebo [27].

**Fig. 12:**
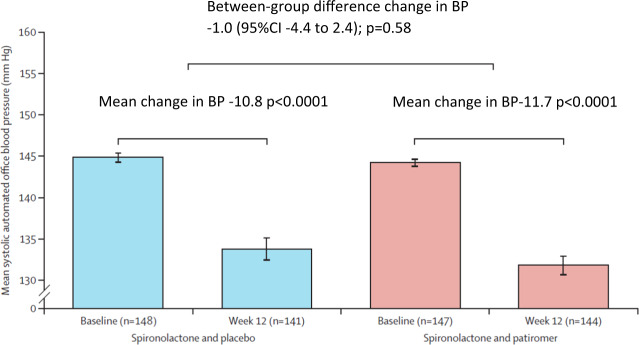
Automated, unobserved systolic blood pressure. Patiromer enables the administration of spironolactone in more patients. However, the use of patiromer in addition with spironolactone was not associated with a significant reduction of blood pressure after 12 weeks [27].

The oral potassium binder patiromer (a non-absorbable potassium-binding polymer with calcium as an exchange ion) lowers serum potassium levels and thus may be useful in patients with medication-related hyperkalemia. In the AMBER trial [27], patiromer was compared with placebo in 295 patients with therapy-resistant hypertension who were treated with three or more antihypertensive substances. The estimated glomerular filtration rate (eGFR) was 25–45 ml/min/1.73 m^2^, and the serum potassium between 4.3 and 5.1 mmol/L. All patients received spironolactone (25–50 mg) and patiromer (8.4 g) or placebo. After 12 weeks, therapy with spironolactone could be continued in 66% of the patients in the control group and 86% of the patients in the patiromer group (group difference 19.5%, 95% CI 10.0–29.0; *p* < 0.0001) (Fig. 11). The cumulative dose of spironolactone was 385 mg higher in the patiromer group (*p* = 0.0021). The side effects were comparable between the two groups. Although a higher dose of spironolactone was administered over a longer period in the patiromer arm, the BP change was not statistically significant between the groups (−1.0 mmHg; 95% CI −4.4–2.4 mmHg; *p* = 0.58) (Fig. 12).

##### Clinical implication

Compared with placebo, patiromer enables administration of spironolactone in more patients with therapy-resistant hypertension and renal insufficiency. However, the use of patiromer in addition with spironolactone was not associated with improved BP control. The long-term effects of a therapy with patiromer (>12 weeks) are unknown.

#### Chronotherapy

**Fig. 13:**
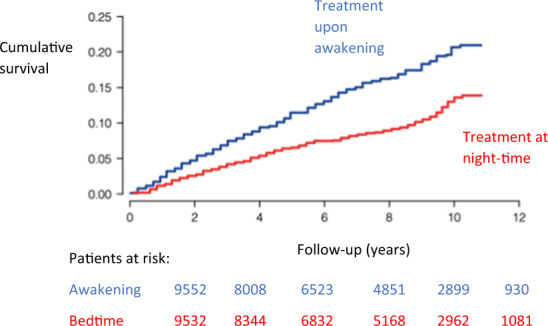
Survival of the two groups. Kaplan–Meier curve for the combined endpoint (cardiovascular death, myocardial infarction, coronary revascularization, heart failure and stroke) comparing treatment upon awakening and at night-time [28].

The European Guidelines [2] do not recommend a preferred administration-time. The Hygia Chronotherapy trial tested whether night-time therapy in comparison to usual upon awakening hypertension therapy exerts a favorable CV risk reduction. The largest study in this context involved >40 Spanish care centers, which included a total of 19,084 hypertensive patients [28]. During an average follow-up of 6.3 years, 1752 participants experienced the primary cardiovascular outcome consisting of CV death, myocardial infarction, coronary revascularization, heart failure, or stroke. An ambulatory BP measurement was performed for 48 h (!) to collect data on how BP differed during sleep. Patients were randomized 1:1 to ingest their entire daily dose of ≥1 hypertension medication at night-time or all of them upon awakening. The RR reduction for various CV events was significantly improved for night-time treatment when compared with awakening treatment: 56% for CV death (HR 0.44; 95%CI 0.34–0.56), 34% for myocardial infarction (HR 0.66; 95% CI 0.52–0.84), 40% for coronary revascularization (HR 0.60; 95% CI 0.47–0.75), 42% for heart failure (HR 0.58; 95% CI 0.49–0.70) and 49% for stroke (HR 0.51; 95% CI 0.41–0.63) (Fig. 13). Interestingly, the reduction of BP was only marginally greater in the night-time group (office SBP: 140 vs. 143 mmHg, *p* < 0.001; office DBP: 82.4 vs. 81.4 mmHg, *p* < 0.001; night-time SBP 118 vs. 114.7 mmHg, *p* < 0.001). There were also significant differences regarding the substances used in the groups (see Table 3), as this was not specified in the study protocol. The limitations of the Hygia study include the PROBE (prospective, randomized, open, blinded endpoint) design, the difference in drug therapy across groups, and the minimal difference in BP between the groups, which can barely account for the enormous effects on outcomes observed. Furthermore, treated and untreated patients were included in the study without differentiation or separate analysis of these subgroups. Moreover, there was no reporting of the preliminary results of the trial before publication and supplementary material providing more details of the protocol and the full data analysis are lacking. Indeed, some experts suggested from studying the protocol that no proper randomization had been performed and the database appeared as a summary database of multiple smaller studies already completed, such as the MAPEC study [29]. Despite the difficulties associated with 48 h of ABPM, only 607 (3%) patients were excluded due to inappropriate measurements. Of note, other studies were unable to demonstrate the superiority of chronotherapy (e.g. AASK [30]).

**Table 3:** Antihypertensive therapy at the end of the examination between groups [28].

Variable	Awakening	Night-time	*P* between groups
Participants, *n*	9552	9532	
Hypertension treatment			
Number of Medications	1.80 ± 0.89	1.71 ± 0.93	<0.001
ARB, %	53.1	53.1	0.995
ACE,I %	25.3	23.4	0.002
CCB, %	32.7	36.8	<0.001
BB, %	22.0	17.5	<0.001
Diuretics, %	46.5	39.5	<0.001

##### Clinical implication

The Hygia study represents the largest study that tested night-time antihypertensive treatment. In this trial, chronotherapy was associated with a significant reduction in endpoints (including death). However, there are other studies that did not show an advantage of the chronotherapy. There are also major limitations in the study design which questions the transfer of the results into routine clinical practice based on the trial’s results alone.

#### Renal denervation

**Fig. 14:**
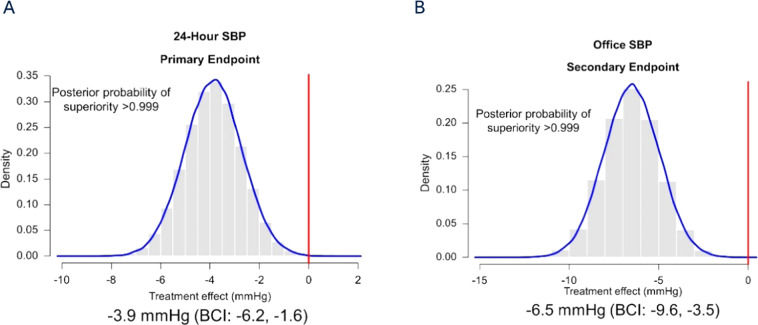
Primary and secondary endpoint after renal denervation. Posterior distribution of between-group differences in (**a**) primary efficacy endpoint (24-h systolic blood pressure) and (**b**) secondary effectiveness endpoint (office systolic blood pressure). BCI Bayesian 95% credible interval, SBP systolic blood pressure [35].

After early clinical trials [31, 32] and registries [33] of renal denervation showed promising results for the therapy to reduce BP, the first sham-controlled SYMPLICITY HTN-3 [34] trial did not prove superiority for the BP-lowering efficacy of renal denervation due to flaws in trial design and conduct. The second-generation of sham-controlled trials were carefully designed to overcome these limitations. The multicenter SPYRAL HTN-OFF-MED [35] and HTN-ON MED [36] trials randomized patients with combined systolic-diastolic hypertension in the absence or presence of antihypertensive medication to renal denervation  using the multielectrode Spyral radiofrequency catheter or a sham procedure. Both trials confirmed the feasibility and safety of the procedure but were not prospectively powered for efficacy endpoints. Recently, the results of the SPYRAL HTN-OFF-MED Pivotal trial, which combined the data of the pilot and pivotal trials using a Bayesian design, were published. The trial was powered for change in mean 24-h and office SBP between baseline and three months. While mean 24-h SBP did not change in the sham group (−0.6 mmHg; 95% CI −2.1 to 0.9), there was a significant reduction in the renal denervation group across 24 h (−4.7 mmHg; 95% CI −6.4 to −2.9). The primary endpoint of a significant between-group difference of change in mean 24-h SBP from baseline to 3 months (−3.9 mmHg; Bayesian 95% credible interval: −6.2 to −1.6) was met (Fig. 14). After the RADIANCE-HTN SOLO trial, which investigated the ultrasound-based Paradise catheter, the SPYRAL Pivotal trial is the second trial powered for efficacy showing the superiority of renal denervation over sham. The procedural safety was also confirmed by a meta-analysis of 50 trials investigating radiofrequency-based renal denervation, which in total included 5769 subjects with 10,249 patient-years of follow-up, but identified only 26 (0.45%) patients with renal artery stenosis or dissection [37].

##### Clinical implication

The SPYRAL HTN-OFF MED Pivotal trial demonstrated the superiority of catheter-based renal denervation compared with sham control to safely lower BP in the absence of antihypertensive medications.

### Summary table

- SBP and DBP are independently associated with myocardial infarction and stroke, although SBP has a greater prognostic effect.
- Studies show an association between hypertension and valvular heart disease outcomes. It remains to be seen how BP control may affect the incidence of heart valve disease progression.
- Twenty-four hours and night-time BP measurements were associated with greater risks of mortality and a composite of CV outcome. Thus, they may be considered as the most relevant measurements for estimating CV risk.
- The overall reproducibility of masked and white-coat hypertension is low and suggests that regular BP examinations (using different measurement modalities) are necessary especially in these patients.
- BP values ≤140/90 mmHg may be associated with an increased risk of mortality in octogenarians or elderly patients with previous cardiovascular events.
- The risk-benefit assessment of an intensified BP treatment should be critically and individually evaluated, especially in older patients.
- Compared with placebo, patiromer enables the administration of spironolactone in more patients with therapy-resistant hypertension and renal insufficiency.
- However, the use of patiromer in addition with spironolactone was not associated with improved BP control.
- The Hygia study represents the largest study that tested night-time antihypertensive treatment. In this trial, chronotherapy was associated with a significant reduction in endpoints (including death). However, there are other studies that did not show an advantage of the chronotherapy. There are also major limitations in the study design which question the transfer of the results into routine clinical practice based on the trial’s results alone.
- SPYRAL HTN-OFF MED Pivotal trial demonstrated the superiority of catheter-based renal denervation compared with sham control to safely lower BP in the absence of antihypertensive medications.
